# Association between the Exposure to Phthalates and the Risk of Endometriosis: An Updated Review

**DOI:** 10.3390/biomedicines12081932

**Published:** 2024-08-22

**Authors:** Bárbara Ribeiro, Melissa Mariana, Margarida Lorigo, Denise Oliani, Ana Cristina Ramalhinho, Elisa Cairrao

**Affiliations:** 1 Faculty of Health Sciences (FCS), University of Beira Interior (UBI), 6200-506 Covilhã, Portugal; a39360@fcsaude.ubi.pt (B.R.); margarida.lorigo@gmail.com (M.L.); 2 Health Sciences Research Centre (CICS), University of Beira Interior (UBI), 6200-506 Covilhã, Portugal; melissa.r.mariana@gmail.com; 3 Faculty of Sciences (FC), University of Beira Interior (UBI), 6201-001 Covilhã, Portugal; 4 Assisted Reproduction Laboratory, Academic Hospital of Cova da Beira, 6200-251 Covilhã, Portugal; vaz.oliani@gmail.com; 5 São José do Rio Preto School of Medicine, Gynaecology and Obstetrics, São José do Rio Preto 15090-000, Brazil; 6 Cova da Beira Local Unit of Health, 6200-251 Covilhã, Portugal

**Keywords:** endocrine disruptors, phthalates, endometriosis, female fertility, epidemiology

## Abstract

Endometriosis is a chronic gynecological disease, primarily associated with pelvic pain and infertility, that affects approximately 10% of the women of reproductive age. Estrogen plays a central role in endometriosis, and there is growing evidence that endocrine disruptors, such as phthalates, may contribute to its development. This review aimed to determine whether there is a causal relationship between phthalate exposure and the development of endometriosis, as well as the possible effects of phthalates on fertility, by analyzing epidemiological data. After a literature search with a combination of specific terms on this topic, we found that although there are limitations to the current studies, there is a clear association between phthalate exposure and endometriosis. Phthalates can interfere with the cellular processes of the endometrium; specifically, they can bind to PPAR and ER-α and activate TGF-β, promoting different signaling cascades that regulate the expression of specific target genes. This may lead to inflammation, invasion, cytokine alteration, increased oxidative stress, and impaired cell viability and proliferation, culminating in endometriosis. Nevertheless, future research is important to curb the progression and development of endometriosis, and strategies for prevention, diagnosis, and treatment are a priority. In this regard, public policies and recommendations to reduce exposure to phthalates and other endocrine disruptors should be promptly implemented.

## 1. Introduction

Endometriosis is a chronic gynecological disease that affects women of reproductive age. It is characterized by the abnormal growth of endometrial tissue in extrauterine locations, particularly in the ovaries, fallopian tubes, and pelvic cavity [1,2]. The spectrum of presentation varies from small superficial lesions to cystic lesions, which may lead to numerous symptoms including chronic pelvic pain, dysmenorrhea, dyspareunia, and infertility [3]. It is a disease that affects approximately 10% of women of reproductive age, corresponding to approximately 190 million women globally [4] and is found in 35–50% of infertile women [1,5]. It is a complex condition that can have a major impact on women’s physical and mental health, affecting their familiar, social, and economic lives and, consequently, society as a whole [6].

It is currently known that the etiology of endometriosis depends on the combined effect of immunological, hormonal, genetic, and environmental factors [7]. Accordingly, several theories for this disease’s pathogenesis have been proposed, including coelomic metaplasia and mullerian remnants hypotheses; hematogenous and lymphovascular dissemination; and stem cell, genetic, and epigenetic theories, but the most widely accepted is the retrograde menstruation theory [1].

In endometriosis, estrogen plays a central role, regulating the main pathological processes. Therefore, considering the increased exposure level to pollutants and the role of endocrine disrupting compounds (EDCs), there is increasing evidence suggesting that the exposure to these toxicants may contribute to the development of this condition [8].

The Endocrinology Society defines EDCs as substances present in the environment, food, and consumer products that interfere with hormone biosynthesis, metabolism, or action, resulting in a deviation from hormonal homeostasis [9]. Phthalates are a class of EDCs widely used as plasticizers and additives in consumer and industrial products [10]. As they are not covalently linked to these products, phthalates are likely to enter the atmosphere, soil, and food, significantly increasing their potential to contaminate the environment. Ingestion, skin absorption, and inhalation are common routes of phthalates’ exposure [11], which, after entering the body, are metabolized to monoesters and subsequently excreted in the urine [12].

Due to their estrogenic properties, phthalates have the ability to compete for endogenous receptors, which can lead to altered hormone levels and affect estrogen-dependent tissues, as in endometriosis [13]. Therefore, several studies have associated phthalates and their metabolites with endocrine disorders and reproductive dysfunctions, including endometriosis [12].

This work aimed to determine whether there is a causal relationship between the exposure to phthalates and the development of endometriosis through a review of human epidemiological data. To conduct this review, a literature search was carried out of different databases, such as PubMed, Elsevier, and Web of Science, using the following combination of key terms: phthalic acid OR phthalate OR phthalates OR phthalate metabolites AND endometriosis OR endometrioses OR endometrioma.

## 2. Endometriosis

Endometriosis is a gynecological, benign, inflammatory, chronic, and hormone-dependent disease defined by the growth of endometrial-type tissue in extrauterine locations, most commonly in the pelvic cavity, namely, in the ovaries, fallopian tubes, uterine ligaments, and the pouch of Douglas; very rarely, it can extend to the colon, lungs, and even the brain [2,3]. Ectopic tissue has morphological and functional characteristics similar to the endometrium—glands and endometrial stroma—which undergoes cyclical changes, leading to cyclical symptoms and chronic inflammation associated with estrogen [14]. This disease occurs in approximately 10% of women of reproductive age, corresponding to around 190 million women globally [3,4] and is found in 35–50% of infertile women [15]. Despite being a common disease, endometriosis is underdiagnosed and undertreated, with an gap of 8 to 12 years between the onset of symptoms and a definitive diagnosis. This is due to the nonspecific nature of the symptoms and the lack of noninvasive diagnostic tests that can confirm the diagnosis [16].

Endometriosis varies greatly in terms of its presentation, clinical manifestation, evolution, response to treatment, and recurrence rate. The spectrum of presentation ranges from superficial lesions and ovarian cysts (endometrioma) to nodules, often accompanied by fibrosis and adhesions (deep endometriosis) [3]. The classic symptoms include chronic pelvic pain, progressive dysmenorrhea, deep dyspareunia, and infertility. However, the disease can present atypically and, in some cases, can be asymptomatic. Furthermore, the severity of the symptoms may not be related to the extent of the disease [17].

Although adequate anamnesis and a positive pelvic examination can indicate the presence of endometriosis, the diversity of the disease’s clinical presentation can make definitive diagnosis challenging. Currently, the gold standard is the direct visualization of the ectopic lesions via laparoscopy, confirmed by a histological study of the biopsy of the lesions. Some imaging methods can be used as first-line examinations, such as transvaginal ultrasound and magnetic resonance imaging. Research has been carried out to find other noninvasive methods, such as biomarkers, to enable earlier diagnosis [3,18]. Staging can be conducted using the American Society for Reproductive Medicine classification, which ranges from stage I, minimal disease, to stage IV, severe disease [19].

### 2.1. Pathophysiology

**Figure 1:**
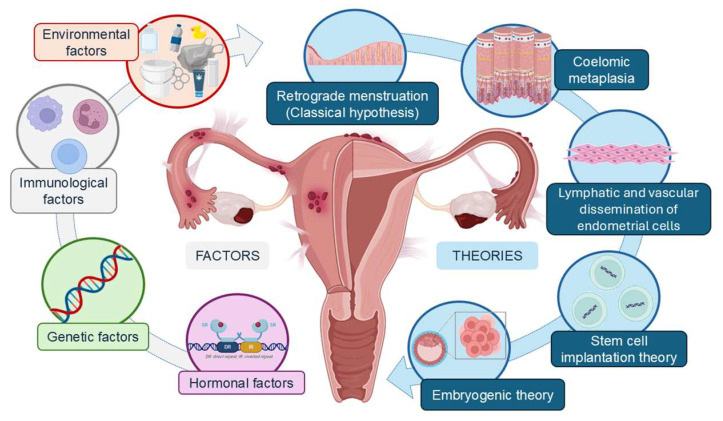
Theories of endometriosis pathophysiology and the main factors involved in its etiology.

Although the pathogenesis of endometriosis is still poorly understood, there are several theories that attempt to explain its origin (Figure 1). The most widely accepted was proposed by Sampson in 1927. The retrograde menstruation theory argues that fragments of the endometrium migrate through the fallopian tubes into the pelvic cavity, where they adhere and proliferate [3]. Other theories have emerged, such as celomic metaplasia, in which the epithelium of the peritoneum undergoes metaplasia and differentiates into cells similar to endometrial cells; the lymphatic and vascular dissemination of endometrial cells; stem cell implantation theory, which states that endometrial and/or hematopoietic stem cells can differentiate into endometrial tissue in different anatomical locations; embryogenic theory, which states that the persistence of residual embryonic cells from Wolff’s or Müller’s ducts can give rise to endometrial tissue in response to estrogen [16,20]. However, none of these theories have definitively demonstrated an absolute causal association with endometriosis, so other factors need to be involved in its etiology [21]. In this sense, a combination of hormonal, genetic, immunological, and environmental factors would allow endometrial tissue to survive and grow outside the uterus [7].

As it is a hormone-dependent disease, pathological endometrial tissue is characterized by the deregulation of estrogen and progesterone. These hormones regulate the main pathological processes in endometriosis, including immunological, inflammatory, angiogenic, antiapoptotic, and cellular processes [8]. Estrogen is produced both systemically and locally by the endometriotic lesions through the increased expression of aromatase and the decreased expression of 17β hydroxysteroid dehydrogenase 2 [22]. The lesions also show increased expression of the β-estrogen receptor, which inhibits apoptosis and promotes proliferation, adhesion, and epithelial–mesenchymal transition. Resistance to progesterone, caused by the suppression of the progesterone receptor and epigenetic changes in related genes, also deregulates the growth and function of the eutopic endometrial lining, with repercussions for fertility [3,21]. There is considerable evidence pointing to the existence of genetic susceptibility to developing the disease, with a greater number of cases occurring in the relatives of affected individuals. In addition, some genetic factors have been identified in genome-wide association studies [23].

Immune system dysfunction leads to an exacerbated inflammatory and immune response, with the production of cytokines, chemokines, and prostaglandins. However, it is unclear whether this dysfunction is responsible for initiating endometriosis or whether it is a characteristic of the disease [3].

Several studies have explored potential epidemiological risk factors associated with endometriosis, including nulliparity, heavy menstrual cycles, early menarche, low body mass index, and the use of oral contraceptives. Infertility, lower general adiposity, and adipose tissue concentrated below the waist have also been reported as high-risk factors. In addition, inconsistent results have been reported on the association of endometriosis with alcohol and caffeine consumption, smoking, and physical activity [7,24]. Due to the complexity of endometriosis, no single factor can fully explain its pathogenesis. It is likely that there is an interaction among these factors that contributes to the development of the disease. Furthermore, it is possible that the influence of each factor varies between women and between each stage of the disease [7].

It is also important to highlight the implications for women’s long-term health, including decreased fertility, increased risk of disease during pregnancy for women who manage to become pregnant [25], followed by the increased risk of cardiovascular and other chronic diseases, as well as possible neoplasms [4].

This evidence supports the view that endometriosis is more than just a gynecological and reproductive disease and highlights the importance of multidisciplinary care involving gynecologists, surgeons, psychologists, radiologists, physiotherapists, and other health professionals to address all the needs of patients with endometriosis. Endometriosis has a high burden on health systems globally, with direct costs ranging from EUR 1332 to 18,478 per patient per year and indirect costs related to work loss, disability, and reduced productivity ranging from EUR 4174 to 12,854 per patient per year [26].

For all the reasons given above, endometriosis is a complex problem that can have a major impact on a woman’s physical and mental health, affecting their family, social, and economic lives and, consequently, society as a whole [6].

### 2.2. Clinical Symptoms and Diagnosis

**Figure 2:**
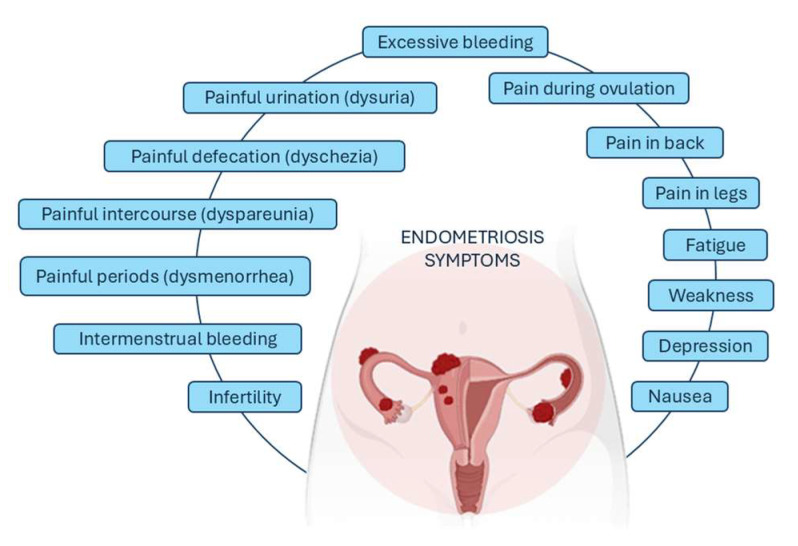
The most common symptoms associated with endometriosis.

The clinical presentation of endometriosis differs from one woman to another. The most frequent symptoms described by patients are intermenstrual bleeding, painful periods (dysmenorrhea), painful intercourse (dyspareunia), painful defecation (dyschezia), and painful urination (dysuria) (Figure 2). Pelvic pain may be present during menstruation or outside this period. Some people also experience heavy bleeding during periods or between periods, trouble getting pregnant, bloating or nausea, fatigue, depression, or anxiety. Symptoms often improve after menopause but not always. Endometriosis can also be asymptomatic, only being detected during evaluation for infertility [18].

The clinical history and physical examination can give significant findings suggestive of endometriosis. Diagnostic imaging methods include multiple modalities such as magnetic resonance imaging (MRI), computed tomography (CT), X-ray, and sonography. Regarding the noninvasive diagnosis of endometriosis, only MRI and sonography in the form of transvaginal ultrasound (TVS) have been proven to be reliable and accurate tools for diagnosing the disease, although surgery is still considered the diagnostic gold standard [27]. As endometriosis is a heterogeneous disease with typical and atypical peritoneal lesions that can range from a single 1 mm peritoneal implant to large endometriomas (10 cm) and cul de sac obliteration, a staging system has been proposed to allow the standardization of the extent of disease. The American Society for Reproductive Medicine classification system for endometriosis (ASRM 1996) is the most widely accepted staging system [19].

The ASRM classifies endometriosis-associated pain symptoms based on the morphology of peritoneal and pelvic implants such as red, white, and black lesions. The percentage involvement of each lesion should be included. For this classification, the pelvis must be inspected in the clockwise or counterclockwise direction. The number, size, and location of endometrial implants, plaques, endometriomas, and adhesions should be noted. Endometriosis in the bowel, urinary tract, fallopian tube, vagina, cervix, skin, or other locations should be documented. According to the ASRM guidelines, endometriosis is classified into stages I (minimal endometriosis), II (mild endometriosis), III (moderate endometriosis), and IV (severe endometriosis) based on the point scores [18].

## 3. Phthalates

Plastic is a constant presence in society. With advances in global industrialization, millions of tons of plastic are produced annually around the world, increasing human exposure to these materials. This constant exposure to plastics has raised human health concerns, especially regarding phthalates.

Phthalates act as EDCs, which are exogenous substances found in the environment, food, and consumer products that interfere with hormone biosynthesis, metabolism, or action, resulting in a deviation from hormonal homeostasis [9,28]. Like hormones, EDCs exhibit specific dose–response curves, represented as U or inverted U curves or without a defined pattern. This means that these compounds may have a powerful effect even at very low concentrations [28]. The mechanisms by which they act are the body is quite complex since, unlike endogenous hormones, they do not have specific receptors, which results in changes in the affinity and specificity of the receptors [29].

Evidence suggests that EDCs can influence the biological or pathological processes related with several diseases such as diabetes and obesity, cardiovascular diseases, different types of cancer (prostate, breast, ovary, uterus), neuroendocrine functions, as well as various gynecological diseases (primary ovarian insufficiency, premature thelarche, endometriosis) [12,29,30].

Since the 1930s, phthalates have been widely used in the industry to impart flexibility, malleability, and elasticity to rigid polymers such as polyvinyl chloride (PVC). Currently, they are present in a wide range of consumer products including cosmetics, food packaging, medical devices, paints, toys, car parts, building materials, hygiene products, and cleaning detergents, among others [10,31,32].

### 3.1. Physical and Chemical Properties

**Figure 3:**
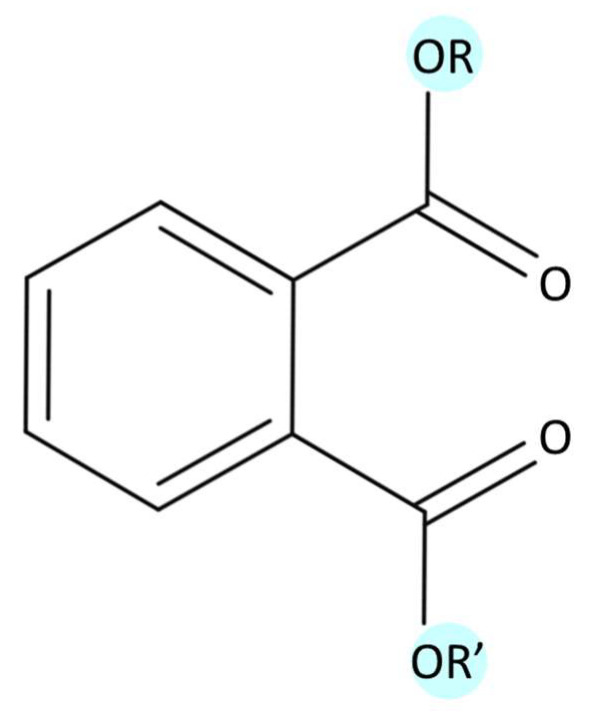
General chemical structure of phthalates (MolView v2.4).

Phthalates are phthalic acid alkyl diesters obtained from the reaction between phthalic anhydride and oxo alcohols, resulting in the formation of esters. Their chemical structure is composed of two aliphatic ester groups linked to one benzene ring (Figure 3) [33]. At room temperature, phthalates are generally colorless and odorless, presenting different physical–chemical properties according to the length of the side chains. Specifically, as alkyl chain length increases, there is decreases in water solubility and vapor pressure, while there is increases in the air–water and octanol–water partitioning coefficients (K_AW_ and K_OW_) [34,35,36]. According to these properties, phthalates with higher molecular weights are highly hydrophobic and more likely to bioaccumulate and biomagnify, showing increased toxicity potential compared to those with lower molecular weights [36]. Thus, phthalates can be classified in two groups: low-molecular-weight (LMW) and high-molecular-weight (HMW) phthalates, as described in Table 1 [12,37].

The most commonly used LMW phthalates are dimethyl phthalate (DMP), diethyl phthalate (DEP), di-n-butyl phthalate (DBP), di-iso-butyl phthalate (DiBP), and butylbenzyl phthalate (BBzP) [32,36]. These compounds are frequently used as solvents in cosmetics, perfumes, creams, candles, shampoos, adhesives, and paints. HMW phthalates are mainly used in construction, toys, bottles, and food packaging. Examples include di-2-ethylhexyl phthalate (DEHP), di-n-octyl phthalate (DnOP), diisononyl phthalate (DiNP), and diisodecyl phthalate (DiDP) [32,36]. DEHP, the most common phthalate used as a plasticizer, is also a component of synthetic leather, waterproof clothing, and numerous plastic medical devices such as blood storage bags and hemodialysis instruments [32,36].

### 3.2. Exposure

The most worrisome phthalate-containing products for human health are cosmetics, food, and medical devices, due to their increased or unavoidable use. Specifically, cosmetics and personal care products are the main sources of human exposure to LMW phthalates, while PVC polymers, plastics, food packaging, and medical devices are significant sources of exposure to HMW phthalates, such as DEHP [32].

An important property for both LMW and HMW phthalates is that they do not form stable and irreversible bonds with the constituent polymers, being easily released, which promotes their dissemination in the environment. This has resulted in soil, food, and atmosphere contamination and the consequent human exposure through ingestion, inhalation, and skin absorption [11].

Direct contact with high-fat food and food at high temperatures increases the migration of plasticizers from the food packaging, mainly DEHP from plastic or PVC packaging. In addition, oral ingestion can occur through indoor air and dust that contain high concentrations of phthalates, primarily DEHP and DEP. Infants and young children are mostly exposed to dust ingestion due to their tendency to put objects in their mouths, while the diet is the main source for older children and adults [10,11]. Other sources of exposure include pharmaceuticals, especially over-the-counter and enteric-coated pills [10].

Compared with the phthalates absorbed through the digestive tract, those absorbed through the skin can be considered more toxic, since this route of exposure avoids the first phase of metabolism and directly affects systemic circulation [29].

### 3.3. Metabolism

Phthalates are readily metabolized to their respective primary monoesters and then converted to oxidative metabolites in the human body. Specifically, the metabolization of phthalates comprises two phases: hydroxylation and conjugation. In the first phase, phthalates are hydrolyzed into monoesters via the action of the esterases and lipases in the intestine or other tissues. In the second phase, oxidative metabolites are catalyzed by the enzyme UDP-glucuronyl transferase, which forms hydrophilic glucuronide conjugates that are easily excreted in the urine within 24 h [12,32]. The monoesters formed in the first phase can be excreted in the urine and feces without further metabolism, as is the case with LMW phthalates. On the other hand, HMW phthalates can undergo hydroxylation or oxidation, forming oxidative metabolites, which then undergo conjugation. The primary metabolites are considered the biologically active compounds of the respective diesters [32,36].

### 3.4. Biomonitoring

The ability to accurately measure exposure to phthalates is a recognized challenge. The quantification of phthalate exposure levels is performed through their corresponding primary metabolites, which are the biologically active compounds. Phthalates have already been measured in the urine, blood, semen, feces, meconium, breast milk, and saliva. Additionally, phthalates can cross the placental barrier and have been quantified in amniotic fluid [10].

The quantification of monoesters in urine is currently the most reliable biological matrix. Urine samples are easy to collect, noninvasive, and reflect the exposure to phthalates over weeks or months, and metabolite levels are generally higher in urine than in other samples. However, considering that HMW phthalates have many oxidative metabolites, the exposure to them is usually estimated by summing the levels of the primary and secondary metabolites in urine [10,36]. Some studies have stated that a single urine sample is sufficient for measuring long-term phthalate exposure [38]; however, considering the short half-lives of phthalates, this may not be the most accurate method. Thus, it has been suggested that using multiple spot urine samples over time from the same individual would reduce the inaccuracy in exposure classification. The temporal stability varies significantly between low- and high-molecular-weight phthalates, being greater for low-molecular-weight phthalates [12,36].

Measuring the phthalate levels in human serum provides information about circulating levels, but blood collection is invasive, the samples are prone to contamination, and the blood that can be collected is generally limited in quantity. Saliva is simpler and noninvasive to collect, and breast milk can be valuable for assessing long-term exposure. However, all of these biological matrices are prone to contamination. Thus, to prevent this from happening, phosphoric acid is used for serum and breast milk samples, while presorted glass tubes are used for saliva. Considering that the serum and saliva levels of phthalates are similar, saliva is preferred due to the noninvasive nature of its collection method [12,36].

The biomonitoring of phthalates is an essential tool for assessing human exposure to these widely used chemical compounds. Given the prevalence of phthalates in the environment and their association with health risks, it is crucial to continue to investigate and improve the methods for detecting and quantifying these compounds in different biological matrices, with urine currently being the most accurate matrix, despite its limitations for temporal detection.

## 4. Phthalates Effects in Endometriosis

Since the chemical structure of phthalates is a similar to that of sex hormones, the reproductive system particularly vulnerable to interference from phthalates. There has been progressive increases in infertility rates in developed countries, often attributed to social and economic changes. However, there is growing evidence that lifestyle changes have increased the exposure to EDCs, such as phthalates, contributing to the emergence of reproductive health problems [29].

Several studies have looked into the possible relationship between exposure to phthalates and endometriosis, with the aim of understanding the impact of these endocrine disruptors on women’s reproductive health.

Acute exposure to bis(2-ethylhexyl) phthalate (DEHP) has been shown to increase cell viability, oxidative stress, and invasive capacity, altering the inflammatory response and estrogen signaling in endometrial cells; these dysfunctions are shared by both eutopic and ectopic endometrial cells in endometriosis [39,40,41].

One of the first studies on the effects of phthalates on endometrial cells revealed that DEHP and MEHP increase the secretion of prostaglandin F2-α (PGF2-α) and decrease the secretion of prostaglandin E2 (PGE2) [42].

Kim et al. demonstrated increased viability and resistance to toxic levels of hydrogen peroxide in a study on cell viability carried out on Ishikawa cells treated with DEHP and endometrial stromal cells. This supports the pathological theory that, by exposing endometrial cells to DEHP, their viability can be increased, thus contributing to the development of endometriosis outside the uterus [39].

Oxidative stress is one of the pathological mechanisms associated with endometriosis [14]. Cho et al. observed an increase in the peroxide-sensitive fluorescent marker level in endometrial stromal cells exposed to DEHP, which indicates the formation of reactive oxygen species (ROS). They also found a decrease in the expression of antioxidant enzymes such as superoxide dismutase (SOD), glutathione peroxidase (GPx), heme oxygenase (HO), and catalase (CAT), which act to prevent the damage caused by ROS [40]. This study also clarified the molecular mechanism that leads to oxidative stress by identifying increases in the mitogen-activated protein kinase (MAPK) and nuclear factor kappa B (NF-κB) signaling pathways after exposure to DEHP. Additionally, since DEHP treatment increased the expression of estrogen receptor-α (ER-α) mRNA in the cells, the study suggested that the change in ER-α caused by DEHP may be the initial mediator of MAPK/NF-κB signaling.

The activity of matrix metalloproteinases (MMPs) is believed to play an important role in the early stages of endometriosis. MMPs are enzymes that regulate the physiological changes in the endometrial cycle through vascular formation and remodeling, and their activities are known to increase in endometriosis [14]. Several studies have confirmed that the expressions of MMP-2 and MMP-9 increase in the ectopic and eutopic endometrial tissues of patients with endometriosis [14] (15, 34). In 2015, Kim et al. demonstrated that MMP-2 and MMP-9 activities were significantly increased in endometrial cells treated with DEHP [41]. In addition, they found that exposure to DEHP increased the phosphorylation of extracellular-signal-regulated kinase (Erk) and the expression of p21-activated kinase-4 (Pak-4). Both biological indicators are related to endometriosis by increasing cell proliferation and resistance to apoptosis [43].

Phthalates, such as DEHP, bind to the peroxisome proliferator-activated receptor (PPAR) and form a pair with the retinoid X receptor (RXR) in the cytoplasm; this pair then translocates to the nucleus and regulates gene transcription [44]. In 2016, Huang et al. associated DEHP with the expressions of interleukin-1β (IL-1β), interleukin-8 (IL-8), MMP2, intercellular cell adhesion molecule-1 (ICAM-1), cyclooxygenase-2 (COX2), and PPARγ, stimulating an inflammatory response that may be mediated by PPARγ [45].

**Figure 4:**
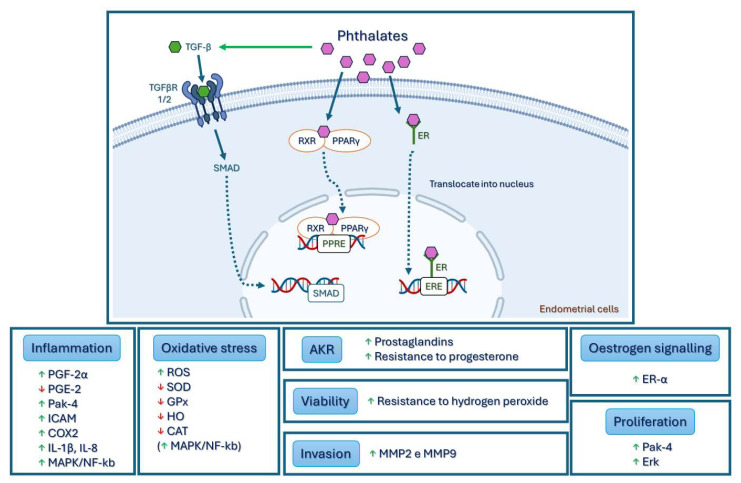
The effect of phthalates on endometrial cells. Phthalate enters cells and binds to receptors such as PPAR and ER-α. By forming complexes with these receptors, it translocates to the nucleus, regulating gene transcription in target genes. Phthalate can also activate TGF-β, which binds to the receptors—TGFβR 1/2—phosphorylating and activating Smad, which are transported to the nucleus as transcription factors to regulate gene expression. After phthalate stimulation, endometrial cells show inflammation; invasion; cytokine alteration; increased oxidative stress, cell viability, and proliferation. Legend: TGF-β—transforming growth factor-β; TGFβR 1/2—TGF-β receptor type 1/2; RXR—retinoid X receptor; PPARγ—peroxisome proliferator activated receptor γ; EREs—estrogen response elements: PPREs—peroxisome proliferator response elements; PGF-2α—prostaglandin F2-α; PGE-2—prostaglandin E2; Pak-4—p21-activated kinase-4; ICAM—intercellular cell adhesion molecule; COX2—cyclooxygenase-2; IL 1β/8—interleukins 1β/8; MAPK—mitogen-activated protein kinase; NF-κb—nuclear factor kappa B; ROS—reactive oxygen species; SOD—superoxide dismutase; GPx—glutathione peroxidase; HO—heme oxygenase; CAT—catalase; Erk—extracellular-signal-regulated kinase; MMP 2/9—metalloproteinase 2/9.

Human endometrial cells from the eutopic endometrium of patients with endometriosis showed increased expressions of aldo-keto reductase (AKR) 1C1, AKR1C2, AKR1C3, and AKR1B10 after exposure to DEHP, so they may play a role in the development of endometriosis through the resistance to progesterone and prostaglandin synthesis [46]. As described in the pathophysiology of endometriosis, epithelial–mesenchymal transition has been suggested to play a significant role in this disease. In a recent study in 2022, the authors showed that DEHP increased proliferation, migration, and inflammation, as well as induced epithelial–mesenchymal transition; they also found the stem cell phenotype in human endometrial and endometriotic cells. The selective inhibitor of transforming growth factor-β (TGF-β) type 1/2 receptor reversed the aforementioned characteristics triggered by DEHP, indicating that the phenomena triggered by DEHP may occur through the TGF-β/Smad signaling pathway [47]. The various mechanisms of action of phthalates are shown in Figure 4.

### Epidemiological Studies

The relationship between the exposure to phthalates and the risk of developing endometriosis has been the focus in several studies.

In 2003, significantly higher plasma concentrations of DEHP were found in patients with endometriosis compared to those in control groups, and 92.6% of the patients had detectable levels of DEHP and MEHP in the peritoneal fluid obtained during surgery [48]. Later, in a case–control study conducted by Reddy et al., the plasma concentrations of phthalates in 49 women with endometriosis were compared with those of 38 infertile controls and 21 fertile controls. They found that the concentrations of DnBP, BBzP, DnOP, and DEHP were higher in patients with endometriosis than in women without endometriosis, regardless of the degree of infertility or proven fertility. Furthermore, a correlation was observed between the concentrations of these phthalates and the severity of endometriosis [49]. Additional studies, both carried out in India, have reported similar results [50,51].

The levels of phthalate esters were also analyzed in the plasma of Korean women with advanced endometriosis (stages III and IV) and in that of women without endometriosis. The authors of this study observed that MEHP levels were significantly higher in the patients with endometriosis than in the controls. However, the association between DEHP and endometriosis was weak, and no significant differences were found in the DEHP and MEHP levels between stages III and IV of the disease [52]. It is worth noting that in the Indian and Korean studies, the cases were recruited from reproductive medicine services and the controls from gynecology services (with other gynecological pathologies), which may involve selection bias [53]. Bearing in mind that, in these studies, phthalates were measured in the plasma and that the samples were collected and analyzed on plastic material, there was a possibility of increasing the phthalate contamination of the samples, leading to potentially misleading results [13]. In fact, in the Cobellis et al. study, no correlation was found between DEHP concentrations and its metabolite, MEHP, suggesting that higher DEHP concentrations in the plasma could be the result of sample contamination. Although the use of disposable glassware can easily avoid this problem, these precautions have only been adopted in more recent studies [54,55]. Pednekar et al., despite using only disposable glassware, used a very small sample (n = 11), compromising the reliability of the results [55]. Nazir et al., on the other hand, analyzed the DEHP levels in women with different stages of endometriosis (stages I–IV) and in fertile women without endometriosis, revealing that serum DEHP levels were higher in patients with endometriosis, especially in advanced stages of the disease [54].

Additionally, several studies have evaluated the relationship between the plasma concentrations of phthalate metabolites and the occurrence of endometriosis. More recently, research has begun to focus on the relationship between endometriosis and phthalate metabolites’ levels in urine, which are considered more sensitive biomarkers of the exposure to phthalate diesters. In Taiwan, two studies have found an increase in the level of urinary MnBP in women with endometriosis [56,57]. In a different study, a twofold higher urinary concentration was found for six phthalate metabolites (MnBP, MCMHP, MECPP, MEHP, MEHHP, and MEOHP) in the population cohort (n = 131) where endometriosis was diagnosed with MRI, while in the operative cohort (n = 495), MOP and MEHP levels were found to be increased during surgery [58]. Additionally, Kim et al. included 55 patients who underwent surgery and were diagnosed with advanced endometriosis, and they found that MEHHP, MEOHP, and MECPP were associated with the disease [41].

In a different approach, Sun et al. collected three different biological samples: blood, urine, and endometrial tissue. The results showed that in the plasma, DBP and DEHP concentrations were higher in women with endometriosis. In the urine, MEHP and ∑DEHP concentrations were significantly increased, while the metabolites MEP, MiBP, MBP, MEOHP, MEHHP, and MECPP had lower concentrations in the endometriosis group. The detection rates of DBP and DEHP in the pathological tissues were similar to those in the plasma, but the concentrations in the tissues were 4 and 14.4 times higher, respectively [59]. Upson et al. identified an inverse relationship between MEHP levels and the risk of endometriosis but observed an increase in the urinary concentrations of MBzP and MEP in women with endometriosis [60]. Similar results were reported by Weuve and et al., where MEHP was significantly higher in the controls than in the cases; that is, there was an inverse association between phthalate metabolites and endometriosis. Similarly, in a different cross-sectional study, a nonsignificant association between MBP and endometriosis was also found [61]. However, the diagnosis of endometriosis in these studies was determined through questionnaires and by recording the history of past illness [61] or confirmed by reviewing records indicating the direct surgical visualization of endometriosis [60]. Given that a definitive diagnosis of endometriosis requires an invasive procedure and histopathological confirmation, the potential for misclassification in these studies is high. Researchers have acknowledged the possibility that the incorrect classification of the disease may have weakened the true associations between phthalate metabolites and endometriosis [13,53].

Three different studies have also found no association between urinary phthalate concentrations and endometriosis [62,63,64]. Itoh et al. found no association between several phthalate metabolites (MnBP, MBzP, MEHP, MEHHP, and MEOHP) and endometriosis in a comparative study between women diagnosed with endometriosis and controls [62]. Similarly, in 2019, in a study conducted in Brazil, no significant differences were observed in the urinary levels of various phthalate metabolites between controls and patients with endometriosis with a confirmed histological diagnosis [63]. Lastly, Zhang et al. described an inverse association between MEHP and the risk of endometriosis; however, in a subanalysis that included only premenopausal women, the authors found a positive association between MiBP and MBzP and endometriosis [64].

The most recent study, carried out in Egypt in 2022, showed that high levels of urinary MEHP were associated with the use of plastic food containers and cosmetics, as well as high levels of estrogen. A significant correlation was also identified between urinary MEHP levels and endometriosis [65] (p. 57).

This discrepancy has also been described in different meta-analyses [66,67,68] and systematic reviews [8,53,69]. However, as is known, this type of review presents some limitations, such as the absence of some studies where the keywords or titles are not well defined or clear. The authors of “Letter to the Editor: Phthalates and Endometriosis” conducted a comprehensive analysis of five existing studies [56,58,60,61,62] and calculated an odds ratio of 0.76, indicating that the overall evidence does not support an association between phthalate concentrations and endometriosis [70]. The discrepancy in the results can be attributed to various confounding factors identified in the studies, such as differences in creatinine adjustment, sample size, study design, phthalate metabolites evaluated, and biological fluids used to measure phthalate concentrations [53,68,69]. From a methodological point of view, many studies can be criticized regarding sample size in general and especially the number of controls in case–control investigations. Considering an endometriosis prevalence of 10% in the population of women of childbearing age, the minimum sample size for obtaining meaningful data should be around 200 individuals [68,69]. Most of the studies mentioned adopted a case–control study design, which has several limitations, including that the associations found may or may not represent causal relationships and the difficulty of establishing true temporality (i.e., whether exposure preceded the outcome or vice versa) [68,69]. Although several in vitro and human studies strongly support the hypothesis that phthalates play a crucial role in the pathogenesis of endometriosis, more studies are needed to understand the mechanism of influence of these EDCs on this disease.

Table 2 summarizes the epidemiological studies mentioned above and their general conclusions.

## 5. Conclusions

The exposure to endocrine-disrupting chemicals, which occurs through various products and routes of contamination, can considerably affect the reproductive health of women. Although the analyzed studies present numerous limitations, from the study design and sample size to the biological fluids and phthalate metabolites evaluated, they suggest an association between the exposure to phthalates and endometriosis.

According to several studies, the potential mechanisms of phthalate esters in endometrial cells involve oxidative stress, inflammatory enzymes, and hormone receptors in cell membranes. It is also assumed that these inter-related signals increase the viability, resistance, and proliferation of endometrial cells. However, many aspects of this relationship are still unknown. Even so, despite the numerous limitations of the studies analyzed, they suggest that the exposure to nonpersistent EDCs, especially phthalates, is associated with endometriosis.

Regarding the epidemiological studies analyzed, most show a positive association between the levels of phthalate diesters and endometriosis, while metabolites show contradictory results, with women with endometriosis showing increased levels in some studies and decreased levels in others. Thus, more studies are needed to understand how phthalates are associated with the development of endometriosis and its severity.

This review highlights the importance of continuing to investigate the relationship between phthalates and endometriosis. Future studies should be conducted with improved methodologies, adequate sample sizes, and more accurate measures of phthalate exposure. In addition, investigating the underlying mechanism through which phthalates influence the pathogenesis of endometriosis is also crucial for better understanding this relationship. Ultimately, the knowledge gained through future research may contribute to a greater understanding of endometriosis and eventually lead to better prevention, diagnosis, and treatment strategies for women affected by this debilitating condition, as well as guide the formulation of public policies and recommendations for reducing the exposure to phthalates and other EDCs.

## Figures and Tables

**Table 1:** LMW and HMW phthalates’ properties and their respective metabolites.

	Phthalate	Chemical Formula	Molecular Weight	Metabolites
LMW	Dimethyl phthalate—DMP	C_10_H_10_O_4_	194.18	Mono-methyl phthalate—MMP
	Diethyl phthalate—DEP	C_12_H_14_O_4_	222.24	Mono-ethyl phthalate—MEP
	Di-n-butyl phthalate—DBP	C_16_H_22_O_4_	278.34	Mono-n-butyl phthalate—MBP Mono-(3-hydroxybutyl) phthalate—MHBP
	Di-iso-butyl phthalate—DiBP	C_16_H_22_O_4_	278.34	Mono-isobutyl phthalate—MiBP
	Butylbenzyl phthalate—BBzP	C_19_H_20_O_4_	312.36	Mono-benzyl phthalate—MBzP Mono-(3-carboxypropyl) phthalate—MCPP
HMW	Dicyclohexyl phthalate—DCHP	C_20_H_26_O_4_	330.42	Mono-cyclohexyl phthalate—MCHP
	Di-2-ethylhexyl phthalate—DEHP	C_24_H_38_O_4_	390.56	Mono-(2-ethylhexyl) phthalate—MEHP Mono-(2-ethyl-5-hydroxyhexyl) phthalate—MEHHP Mono-(2-ethyl-5-oxohexyl) phthalate—MEOHP Mono-(2-ethyl-5-carboxypentyl) phthalate—MECPP Mono-(2-carboxymethylhexyl) phthalate—MCMHP
	Di(2-ethylhexyl) terephthalate—DEHTP	C_24_H_38_O_4_	390.56	Mono-2-ethyl-5-carboxypentyl terephthalate—MECPTP
	Di-n-octyl phthalate—DnOP	C_24_H_38_O_4_	390.56	Mono-n-octyl phthalate—MnOP Mono-(3-carboxypropyl) phthalate—MCPP
	Diisononyl phthalate—DiNP	C_26_H_42_O_4_	418.61	Mono-isononyl phthalate—MiNP Mono-(carboxyisooctyl) phthalate—MCOP
	Diisodecyl phthalate—DiDP	C_28_H_46_O_4_	446.66	Mono-isodecyl phthalate—MiDP Mono-(carboxyisononyl) phthalate MCNP

**Table 2:** Epidemiological studies and their general conclusions.

Author Year	Type of Study	No. of Cases/Control	Biological Sample	Metabolites	General Conclusions
Cobellis et al., 2003 [48]	Case–control	35/24	Blood samples Peritoneal fluid	DEHP, MEHP	DEHP levels were significantly higher in the serum of patients with endometriosis. MEHP levels were low and did not differ between the groups.
Reddy et al., 2006 [49]	Case–control	49/59	Blood samples	BBzP, DEHP, DnBP, DnOP	Phthalate were found in all the samples from women with endometriosis.
Reddy et al., 2006 [51]	Case–control	85/135	Blood samples	BBzP, DEHP, DnBP, DnOP	DnBP, BBzP, DnOP, and DEHP were higher in the endometriosis group and significantly correlated with the severity of endometriosis.
Rozati et al., 2008 [50]	Case–control	99/135	Blood samples	BBzP, DnBP, DEHP, DEP, DMP	DMP, DnBP, BBzP, and DEHP levels were significantly higher in the serum of women with endometriosis and correlated with the severity of endometriosis. DEP was not detected in the study group or in the control group.
Itoh et al., 2009 [62]	Case–control	57/80	Urine	MBzP, MEHHP, MEHP, MEOHP, MEP, MnBP	No significant association was found between the urinary concentration of phthalates and endometriosis.
Huang et al., 2010 [56]	Case–control	28/29	Urine	MMP, MEP, MnBP, MBzP, MEOHP, MEHHP	MnBP levels were higher in women with endometriosis.
Weuve et al., 2010 [61]	Transversal	87/1020	Urine	MBP, MBzP, MEHHP, MEHP, MEOHP, MEP	MBP levels were positively associated (nonsignificantly) with endometriosis. MEHP was inversely associated with endometriosis.
Kim et al., 2011 [52]	Case–control	97/169	Blood samples	DEHP, MEHP	MEHP plasma levels were significantly higher in patients with advanced endometriosis. DEHP had a statistically weak association with endometriosis.
Upson et al., 2013 [60]	Case–control	92/195	Urine	MEHP, MEHHP, MEOHP, MECPP, MBzP, MEP, MiBP, MnBP, DBP	DEHP metabolites, particularly MEHP, may be associated with decreased risk of endometriosis (strong association). MBzP and MEP may be associated with increased risk (no statistical significance). No significant association was found between endometriosis and other DEHP metabolites (MEHHP, MEOHP, or ΣDEHP).
Buck Louis et al., 2013 [58]	Population cohort	14/113	Urine	MnBP, MBzP, MCHP, MCMHP, MCPP, MECPP, MEHHP, MEHP, MEOHP, MEP, MiBP, MMP, MNP, MOP	MnBP, MCMHP, MECPP, MEHP, MEHHP and MEOHP were significantly associated with endometriosis, with a twofold increase in odds.
	Operative cohort	190/283			MOP was associated with endometriosis when restricted to visualized and histological endometriosis. MEHP was associated with endometriosis when the comparison was restricted to women with a postoperative diagnosis of normal pelvis.
Kim et al., 2015 [41]	Cohort	55/33	Urine	MBzP, MECPP, MEHHP, MEOHP, MBP	MEHHP, MEOHP and MECPP levels were significantly higher in women with endometriosis than in the control group.
Sun et al., 2016 [59]	Case–control	134/176	Blood samples	DBP, DEP, DEHP	DBP and DEHP levels were higher in women with endometriosis.
		Urine	MMP, MEP, MiBP, MBP, MEHP, MEOHP, MEHHP, MECPP, MCMHP	MEP, MiBP, MBP, MEOHP, MEHHP, and MECPP levels were significantly lower in the case group. MEHP and ∑DEHP levels were significantly higher in the case group.	
		130/n/a	Endometrial tissue	DnBP, DEP, DEHP	The detection rates of DBP and DEHP in pathological tissues were similar to those in plasma, but the concentrations in tissues were 4 and 14.4 times higher, respectively.
Pednekar et al., 2018 [55]	Case–control	11/34	Blood samples	MMP, MBzP, MEHP, MEHHP	MBzP and MEHHP levels were higher in women with endometriosis. There was no difference in the levels of MMP and MEHP.
Nazir et al., 2018 [54]	Case–control	50/50	Blood samples	DEHP	Higher levels of DEHP were associated with more advanced stages of endometriosis—III and IV. DEHP was not detected in the control group.
Moreira Ferandez et al., 2019 [63]	Case–control	30/22	Urine	MMP, MiBP, MBP, MCHP, MNP, MOP, MBzP, MEHP	No association was found between phthalate metabolites and endometriosis.
Chou et al., 2020 [57]	Case-control	123/82	Urine	MBP, MEHP, MBzP, MEOHP, MEHHP	MnBP levels were higher in the endometriosis group than in the controls.
Zhang et al., 2021 [64]	Transversal	77/1127	Urine	MBP, MBzP, MCPP, MEHP, MEP, MiBP,	In a subanalysis that included only premenopausal women, MiBP and MBzP were positively associated with endometriosis. MEHP had an inverse association with endometriosis.
